# ADAM: advanced design and AI-driven modeling for plant tissue culture media optimization

**DOI:** 10.1186/s13007-026-01534-5

**Published:** 2026-04-26

**Authors:** Hans Bethge, Traud Winkelmann, Tomás A. Arteta, Esmaeil Nezami, Marco Pepe, Mohsen Hesami, Andrew Maxwell Phineas Jones, Mariana Landin, Pedro P. Gallego

**Affiliations:** 1 Institute of Botany, Department of Phytophotonics, Leibniz Universität Hannover, Herrenhäuser Str. 2, 30419 Hannover, Germany; 2 Agrobiotech for Health, Plant Biology and Soil Science Department, Biology Faculty, Vigo University, 36310 Vigo, Spain; 3 Institute of Plant Genetics, Section Reproduction and Development, Leibniz University Hannover, 30419 Hannover, Germany; 4 Department of Plant Breeding, Nuclear Agriculture Research School, Nuclear Science and Technology Research Institute (NSTRI), Karaj, P.O. Box 31485-498, Iran; 5 Department of Plant Agriculture, University of Guelph, Guelph, ON Canada; 6 Pharmacology, Pharmacy, and Pharmaceutical Technology Department, I+D Farma (GI-1645), Faculty of Pharmacy, The iMATUS and the Health Research Institute (IDIS), University of Santiago de Compostela, 15782 Santiago de Compostela, Spain

**Keywords:** Plant tissue culture, Machine learning, Predictive modelling, Evolutionary algorithms, Culture media optimization

## Abstract

**Background:**

Optimization of biotechnological processes is traditionally limited by time-consuming trial-and-error approaches and the complexity of simultaneously optimizing multiple, often conflicting objectives. This applies particularly to plant tissue culture medium design, which therefore serves as the application case in this study. Recent advances in machine learning and evolutionary algorithms offer powerful alternatives, yet 80% of published studies rely on licensed software, and systematic data-driven optimization frameworks remain scarce. This creates significant barriers to adoption in both academic and commercial plant biotechnology.

**Results:**

We introduce ADAM (Advanced Design and AI-Driven Modeling for Plant Tissue Culture Media Optimization), an open-access, web-based platform that transforms protocol development into a data-driven computational process. ADAM implements a complete ML-EA workflow through five integrated modules: 1. Design of Experiments (five different concepts) for systematic parameter exploration, 2. Data Preparation with automated quality control, and 3. Model Building using nine machine learning algorithms with automated selection. The platform enables Optimization (4.) through four advanced evolutionary algorithms (genetic algorithm, particle swarm optimization, NSGA-II, SMS-EMOA) for single- and multi-objective problems, with Evaluation (5.) tools to compare original versus optimized solutions. Validation across two plant tissue culture applications showed that ADAM’s models matched or exceeded the predictive performance of manually optimized approaches in the original studies. The platform successfully identified multiple optimal culture conditions balancing conflicting objectives, providing experimentally testable predictions that reduce the trial-and-error cycle.

**Conclusions:**

Deployed as a browser-based application requiring neither specialized hardware nor software licenses, ADAM democratizes advanced AI optimization for plant biotechnology, eliminating traditional barriers to entry while maintaining the rigor and flexibility required for scientific research.

**Supplementary Information:**

The online version contains supplementary material available at 10.1186/s13007-026-01534-5.

## Introduction

Maximizing performance and economic viability in modern biotechnological processes fundamentally relies on efficient process optimization. This applies in particular to in vitro cell culture approaches, with plant tissue culture protocol development being especially challenging. On the one hand, micropropagation and plant regeneration are complex, multifactorial biological processes where numerous factors such as genetic factors, environmental conditions, and media components interact in non-linear and non-deterministic ways [24, 66, 71]. On the other hand, plant in vitro culture techniques provide powerful tools for plant breeding, propagation, and conservation, and they are essential for implementing modern breeding technologies such as genome editing.

For success in commercial applications, achieving high propagation rates while limiting the occurrences of morpho-physiological disorders and culture decline is crucial [56, 61]. This is largely achieved through the determination and implementation of optimal culture media composition, environmental conditions, and their complex interactions [62]. Traditionally, media optimization relies on laborious trial-and-error experiments, which are both time-consuming and resource-intensive. For example, optimizing just three medium components, each tested at five concentration levels, requires 5^3^ = 125 unique media formulations in a full factorial design. With at least three replicates per treatment, this translates to 375 experimental units—a number that quickly becomes impractical as complexity increases, while plant material is often limited.

This complexity is further compounded by the sheer number of variables involved in medium composition, making medium design both resource-intensive and time-consuming [64]. Hildebrandt et al. [39] estimated that more than 16,000 different treatments were needed to develop a new culture medium. Similarly, Murashige and Skoog [52] took five years to establish their basal culture medium, using eighty-one different combinations of macro- and micro-elements and vitamins. While these important historic works relied on traditional approaches typically adopting a one-factor-at-a-time approach to optimization, more powerful computational approaches can now be employed to critically reduce the number of treatments necessary for optimization of biological responses [61].

This inherent biological complexity results in highly variable responses, presenting significant optimization challenges. Traditional statistical methods and simple mathematical approaches struggle to effectively address these issues, particularly when dealing with noisy, complex, and high-dimensional data [20, 22, 47]. The complex interactions among multiple factors make conventional optimization problematic, often requiring an impractical and unrealistic number of experimental treatments—especially when optimizing for multiple, often conflicting, objectives simultaneously (e.g., maximizing multiplication rates while minimizing growth anomalies) [57]. Consequently, the field requires advanced computational approaches within the domain of Artificial Intelligence (AI) to handle this biological complexity and automate intricate tasks. Data-driven Machine Learning (ML) models offer a powerful alternative by flexibly capturing these complex, non-linear relationships, essentially functioning as surrogate models—intelligent approximations that learn from limited experimental data to predict tissue culture outcomes.

Among computational approaches, Evolutionary Algorithms (EAs) excel at finding near-optimal or satisfactory parameter combinations in complex, multi-objective optimization landscapes. Unlike gradient-based methods that can be trapped in local optima, EAs typically maintain a population of diverse solutions, allowing them to explore multiple regions of the parameter space simultaneously—a crucial feature in tissue culture where optimization landscapes can be highly rugged due to complex factor interactions. However, depending on problem complexity and algorithm configuration, EAs may require hundreds to thousands of function evaluations to converge to good solutions [24]. In tissue culture media optimization, where each evaluation requires weeks of costly laboratory work and resources, this is impractical [19]. ML models address this fundamental limitation by providing a computationally inexpensive approximation of the objective function (here, the relationships between culture media and processing conditions and desired outcomes [57]). These models learn from limited preliminary experimental data, enabling EAs to rapidly explore the vast and uncertain parameter space and intelligently identify promising regions before costly laboratory validation. Using the predictions of ML models transforms optimization from a slow, exhaustive, and resource-intensive experimental process into an intelligent, data-driven search, substantially reducing both time and resource requirements [24, 61].

This ML + EA framework is particularly well-suited for retrospective optimization, where experimental data have already been collected and the goal is to extract maximum predictive insight and identify optimal conditions from existing datasets. An alternative strategy, active learning (AL)—most prominently implemented as Bayesian Optimization with Expected Improvement—is specifically designed for prospective settings where data collection can still be influenced, iteratively selecting the most informative next experiment based on model uncertainty to maximize sample efficiency [53, 73], however, this requires direct control over the experimental sequence, which is not available when working with historical datasets [62].

The application of ML and optimization approaches in plant tissue culture is well-documented in several comprehensive reviews [33, 73]. Optimization can be applied across all phases of plant tissue culture, including optimization of disinfection protocols for culture induction [17, 31, 35, 63], culture media formulation for callogenesis [4, 26, 34, 37, 59], proliferation [11, 22, 36, 55], rooting [9, 20, 27], and environmental conditions for acclimatization [20, 21, 32].

Early applications focused on predictive modeling using neural networks [24, 66] to detailed descriptions of ML models and optimization algorithms [33] and simulations to benchmark optimization algorithms for cell culture media optimization in general [73]. For the general design of experiments (DoE), we refer to Antony [7]. However, to quantify the methodological gaps in this rapidly evolving domain, we conducted a systematic analysis of recent literature (Table 1).

**Table 1:** Literature summary of 50 studies dealing with ML approaches for plant tissue culture media optimization

Optimization	Studies	Years	References
Regression/modeling	20 (40%)	2010–2024	Arteta et al. [11], Arteta et al. [12], Gago et al. [19], Gago et al. [23], Gago et al. [21], 26.García-Pérez et al. [26, 27], Isak et al. [40], Kirtis et al. [45], Lozano-Milo et al. [49], Mridula et al. [51], Nezami-Alanagh et al. [54], Nezami-Alanagh et al. [55], Nezami-Alanagh et al. [56], Niazian et al. [58], Niazian et al. [59], Osama et al. [60], Prakash et al. [65], Şimşek et al. [69], Verma et al. [70]
Single-objective	18 (36%)	2013–2023	Aasim et al. [2], Akin et al. [4], Akin et al. [5], Akin et al. [3], Arab et al. [10], Arab et al. [8], Arab et al. [9], Barone [13], Hesami and Jones [34], Hesami et al. [30], Jafari and Daneshvar [41], Jafari et al. [42], Jamshidi et al. [43], Jamshidi et al. [44], Mehrotra et al. [50], Pepe et al. [63], Saheli et al. [68], Zhang et al. [72],
Single-composite*	4 (8%)	2010–2024	Ali and Aasim [6], Gago et al. [20], Gago et al. [22], Nezami-Alanagh et al. [57]
Multi-objective	8 (16%)	2019–2024	Aasim et al. [1], Gammoudi et al. [25], Hesami et al. [35], Hesami et al. [37], Hesami et al. [37], Hesami et al. [38], Sadat-Hosseini et al. [67], Zarbakhsh et al. [71]

35 were randomly selected studies from the review of Hesami and Jones [33] and the additional 15 articles were published after 2020

*Reducing a multi-objective problem to a single-objective problem

This analysis revealed a clear reliance on basic techniques: out of 50 analyzed studies, 40% relied solely on predictive modeling and statistical analysis without additional optimization techniques. While optimization was addressed in the remaining studies, most avoided the complexity of multi-objective optimization: 36% employed single-objective approaches, and 8% converted multi-objective problems into weighted composite functions. Crucially, only 16% of studies coupled predictive modeling with true multi-objective Meta-heuristic optimization.

**Fig. 1:**
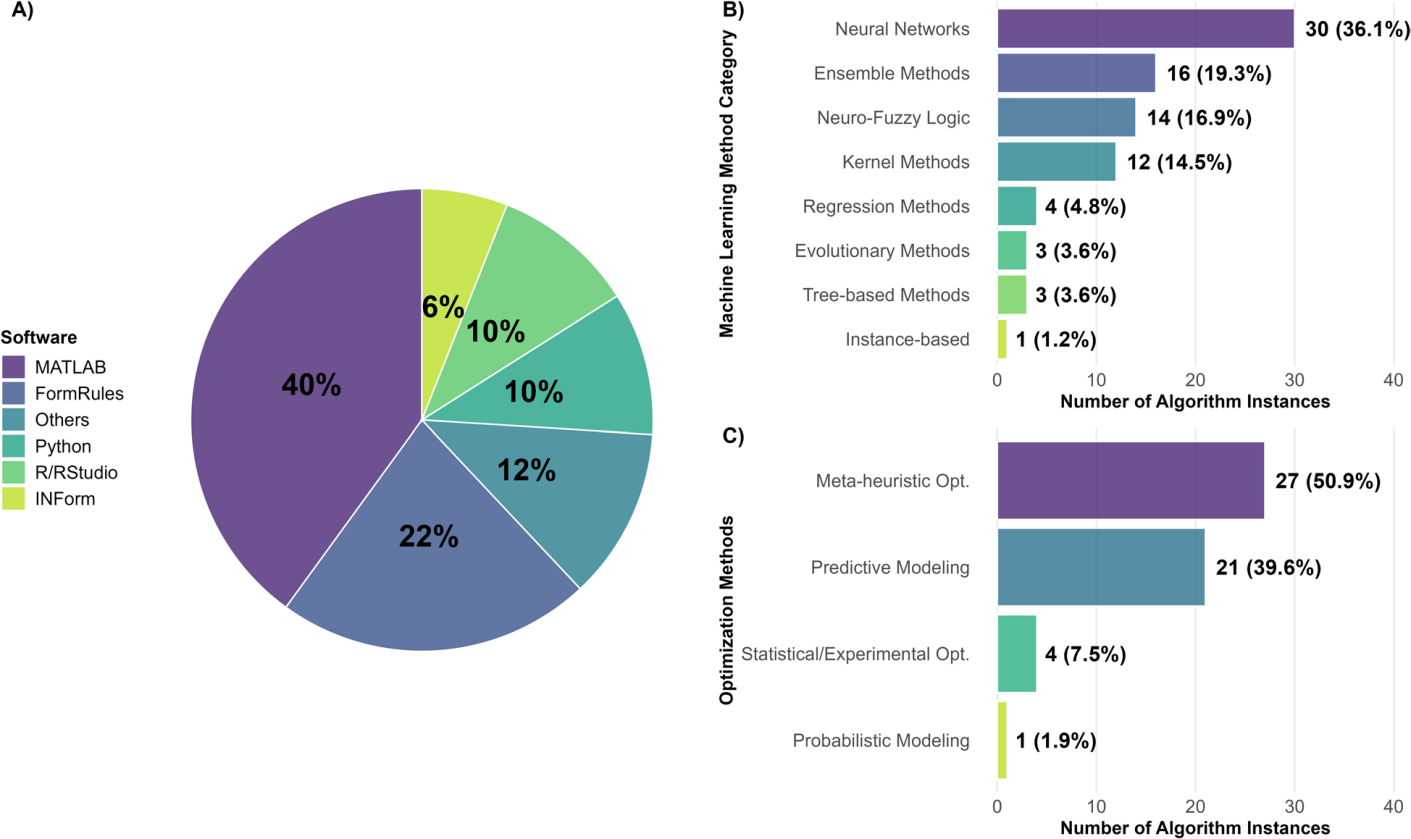
Literature summary of optimization approaches in plant tissue culture. Analysis based on 50 studies listed in Table 1. **A** Software platforms used for optimization, categorized into six groups: MATLAB, FormRules, Python, R/RStudio, INForm, and Others (including SPSS, NeuroSolutions, GeneXpro). **B** ML algorithms used for predicting output variables, categorized into eight methodological groups: neural networks (ANN variants), Neuro-Fuzzy Logic (ANFIS), Ensemble Methods (RF, XGBoost, ESR), Kernel Methods (SVR, GP), Tree-based Methods (CHAID variants), Regression Methods (MLR, MRA, ElasticNet, MARS), Evolutionary Methods (GEP, Symbolic regression), and Instance-based methods (KNN). **C** Optimization algorithms used to find optimized candidate solutions, categorized into five methodological groups: Meta-heuristic optimization (Evolutionary Algorithms: GA, NSGA II; Swarm Intelligence: PSO, FOA; Other Meta-heuristics: BBO, ISA, SOS), and Predictive Modeling without direct optimization (ANNs, ANFIS, SVR among others), Statistical/Experimental optimization (RSM), and Probabilistic Models (HMM). For panel A, each study contributed one instance (n = 50); for panels B and C, multiple instances per study were possible (n = 83 and n = 53, respectively)

This limited adoption highlights the need for integrated, model-based optimization frameworks capable of handing out the inherent complexity and conflicting objectives of in vitro culture protocols. Further analysis of the literature highlights challenges in tool accessibility and algorithm diversity (Fig. 1A). A noteworthy 80% of studies utilized commercial or licensed software, with MATLAB (40%) and FormRules (22%) being most prominent, while other proprietary tools (such as INForm, SPSS, NeuroSolutions, and GeneXpro) accounted for the remaining 18%. In contrast, open-source platforms such as Python and R/RStudio were only used in 20% of the studies. This heavy reliance on licensed software suggests a substantial barrier to entry, underscoring the urgent need for non-licensed, open-access tools to improve accessibility in plant tissue culture protocol optimization. Regarding the underlying technology, neural networks were the most frequently applied ML algorithms for modeling tissue culture traits (36.1% of cases), followed by Ensemble Methods (19.3%) and Neuro-Fuzzy Logic (16.9%). Kernel-based techniques accounted for 14.5%, while classical regression, evolutionary, tree-based, and instance-based methods were used less commonly (Fig. 1B).

Finally, for optimization, Meta-heuristic approaches were the most prevalent, representing 50.9% of cases, with Evolutionary Algorithms (e.g., GA and NSGA-II) being the dominant subtype (41.5%). In comparison, predictive modeling techniques (e.g., ANNs, ANFIS, SVR among others) without direct optimization comprised 39.6% of the approaches (Fig. 1C).

The integration of predictive ML models with optimization algorithms creates a practical and economical means to optimize plant tissue culture systems, given their computational requirements and ease of implementation. However, most platforms that allow this hybrid approach are licensed, underscoring the need for an open-source, web-based alternative with a simple user interface that minimizes the need for programming skills [61]. To directly address these limitations (namely the reliance on licensed software, the omission of multi-objective optimization, and the lack of accessible, integrated workflows) we developed ADAM (Advanced Design and AI-Driven Modeling for Plant Tissue Culture Media Optimization). ADAM tackles the key barriers identified in our literature analysis by providing the following features:

- *Open access*: no licensing barriers, making it open access to research, education, and industry applications.
- *Guided application*: an out-of-the-box browser-based graphical user interface (GUI) that guides users through the process, making advanced ML and optimization techniques accessible to biotechnology professionals and scientists without specialized programming or computational expertise.
- *Complete end-to-end workflow*: covering all necessary steps, from DoE set up to predictive modeling, and the final identification of single and/or multi-objective optimized candidate solutions.
- *Data security and IP protection*: ADAM does not store, log, or make any use of data uploaded by users—all computations are performed transiently within the session, and no user data persists on the server after the session ends, which is critical due to the sensitivity and proprietary nature of culture media recipes and intellectual property. For users requiring full data sovereignty, self-hosting ADAM on local infrastructure is possible upon request by contacting a corresponding author.
- *Reproducibility and transparency*: Support for reproducible workflows, through exportable configurations and logged results, and transparent optimization processes, with interpretable model *outputs*.

To adequately present the utility of ADAM, the current work is organized into three parts: First, we present ADAM’s five-module architecture spanning experimental design, data analysis, modeling, optimization, and evaluation. Second, we validate the platform using two use cases for optimizing plant tissue culture media. Finally, we discuss implications for accelerating biotechnology protocol development and provide complete methodological documentation for reproducibility.

## Software architecture

### Design and implementation of ADAM

**Fig. 2:**
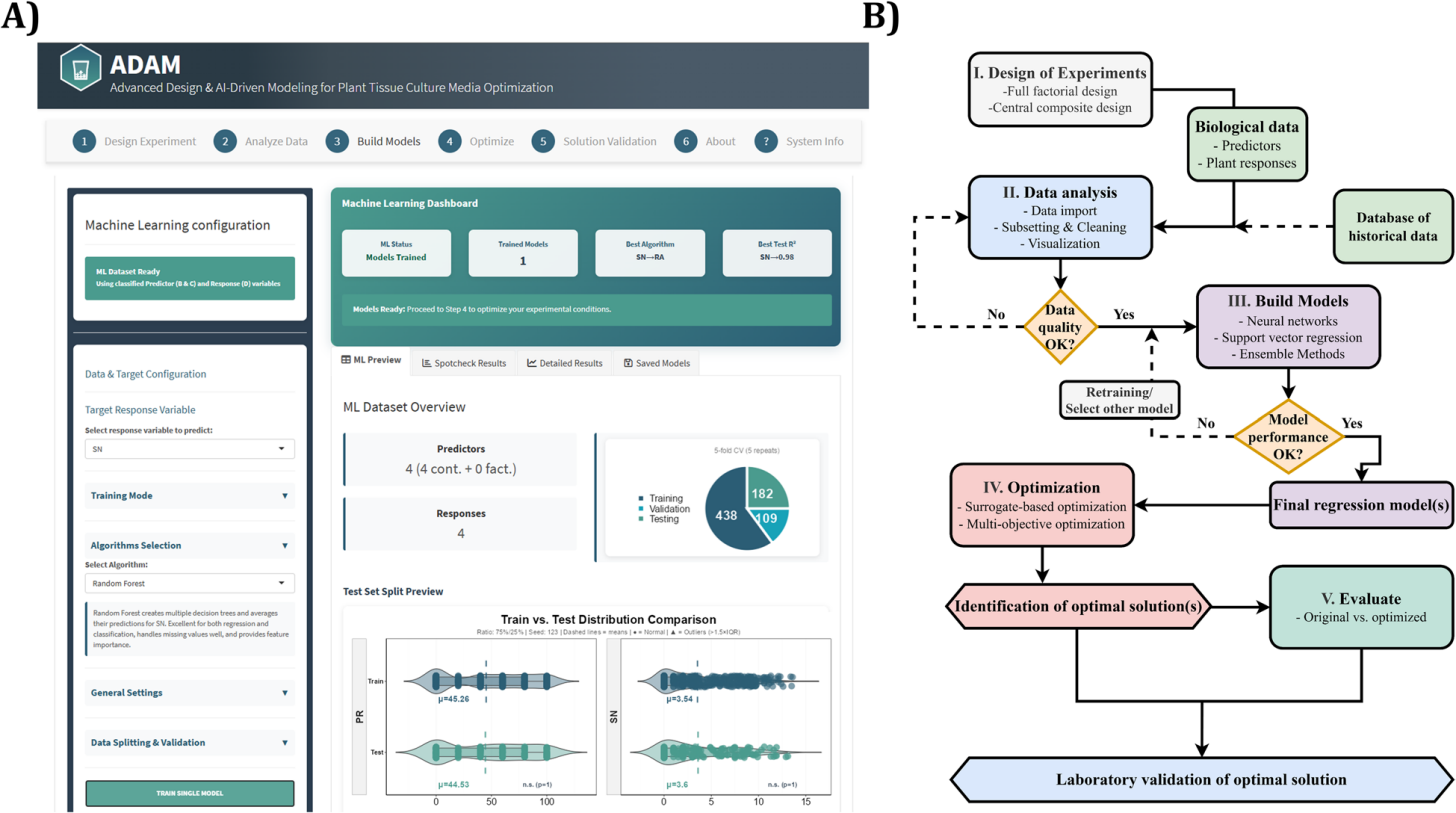
ADAM workflow for ML-driven optimization of biotechnological processes. **A** User interface screenshot showing the machine learning configuration dashboard with dataset overview and model training options. **B** Workflow diagram illustrating ADAM’s five integrated modules: (I) Design of Experiments—supports full factorial, response surface, and space-filling designs for systematic parameter exploration; (II) Data Analysis—encompasses data import, quality assessment, subsetting, cleaning, and visualization with iterative quality control loops; (III) Model Building—trains multiple ML algorithms (neural networks, support vector regression, ensemble methods) with automated hyperparameter tuning and performance validation; (IV) Optimization—applies multi-objective evolutionary algorithms using trained ML models as fitness functions to identify optimal process conditions; (V) Evaluation—compares optimized solutions against original data for validation. The framework includes decision points for data quality and model performance assessment, with iterative retraining pathways to ensure robust predictions before optimization

ADAM integrates a state-of-the-art workflow for ML and evolutionary optimization, specifically tailored for plant tissue applications (Fig. 2A, B). The software is implemented as a web-based application using the R statistical programming language and Shiny framework [16]. ADAM relies on established R libraries: "DoE.base" [29] for experimental design, "rsm" [48] for response surface methodology, "lhs" [15] for space-filling designs, "caret" [46] for ML model training, and "ecr" [14] for evolutionary computation.

#### Design of experiment

The DoE module supports five experimental design methodologies. Users define factors, factor levels, and replicates for their experimental design.

*Full Factorial Design (FFD):* Evaluates all possible factor level combinations (x^k^ runs, where x = levels per factor, k = number of factors). Ideal for complete interaction analysis with small factor numbers.

*Central Composite Design (CCD):* Response surface methodology for studying factor interactions and modeling nonlinear responses. Employs an alpha value (α = 0.8) to ensure factor levels remain within realistic, non-negative limits. The number of center point replications is automatically determined by n₀ = max(4, 2k), where k is the number of factors, ensuring at least four center points for reliable error estimation while scaling with design complexity. Linear transformation steps convert coded coordinates into actual experimental factor levels. Suitable for optimizing continuous factors (nutrients, hormones, pH) within biologically viable ranges.

*Box-Behnken Design (BBD):* Three-level factorial design for quadratic response surfaces without extreme corner points (requires ≥ 3 factors). Suitable when extreme factor levels may cause non-viable culture conditions.

*Latin Hypercube Sampling (LHS):* Space-filling design ensuring uniform coverage of continuous parameter spaces. The algorithm divides each factor’s range (user-defined min/max) into equal-probability intervals, taking exactly one sample per interval. The resulting design maintains the Latin hypercube property, ensuring even exploration of multidimensional spaces. Suitable for broad exploratory studies with even representation across all factor ranges.

*Random Design (RD):* Space-filling random sampling for large experimental domains. For small design spaces (≤50 combinations), RD defaults to full factorial layout. For larger spaces, it selects a manageable subset (typically 20–100 runs or ~30% of all combinations) through random sampling. Strategic points are added including corner points (to test extremes) and center points (to detect response curvature). Appropriate for exploratory studies or high-dimensional problems where structured designs would require too many runs.

A cleanup algorithm identifies and reduces excessive replications (default limit: five per treatment) to prevent computational inefficiency while preserving design properties.

#### Data analysis

The data analysis module ensures data integrity through automated quality assessment, identifying outliers, missing values, and standardizing factors. The core feature is automated data health check, which translates raw experimental results into structured ML-compatible format by guiding users to classify data into four essential variable types:

- *Descriptors (Type A—meta information)*: Experimental metadata including run identifiers, block assignments, replicate numbers, and treatment codes. Automatically detected and excluded from ML training to preserve experimental traceability.
- *Quantitative predictors (Type B—continuous and count inputs)*: Numerical inputs representing medium composition (carbon source, salt and ion concentrations), PGRs, vitamins, and culture environmental conditions (temperature, light intensity, photoperiod, pH). Auto-classification identifies these as continuous predictors suitable for optimization.
- *Categorical predictors (Type C—nominal, binominal and ordinal inputs)*: Categorical experimental factors requiring discrete level selection (genotype, media type, environmental conditions). Auto-classification targets variables with 2–20 unique categorical levels. Categorical levels are integer-encoded prior to ML training (e.g., {"Genotype A", "Genotype B", "Genotype C"} → {0, 1, 2}), assigning a unique integer to each level. During optimization, the algorithm operates in continuous space and Type C variables are rounded to the nearest integer at the candidate evaluation stage—before each candidate is passed to the ML model for prediction—ensuring that only valid category assignments are evaluated by the surrogate model.
- *Response variables (Type D—target outputs)*: Measured experimental parameters (dependent variables) to predict and optimize, including multiplication rate, regeneration rate, shoot height, rooting percentage, root number, plant quality indicators, and economic factors (e.g., media costs). Auto-classification identifies variables with high variability and continuous distributions characteristic of experimental responses.

**Fig. 3:**
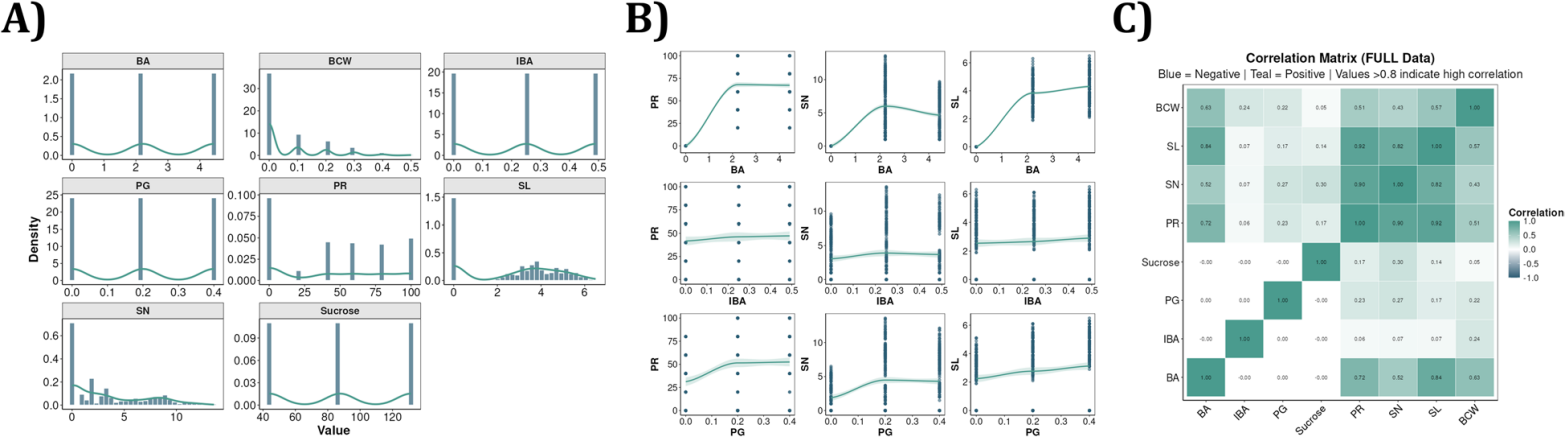
ADAM Data Analysis module visualizations for Use Case I. **A** Distribution charts showing the density and frequency distribution of all predictor and response variables, enabling users to assess data spread, identify discrete dosing patterns, and detect potential outliers prior to model training. **B** Scatter plots with fitted smooth curves illustrating the marginal relationships between key growth regulators (BA, IBA, PG) and the three response variables (PR, SN, SL), supporting exploratory assessment of factor-response associations. **C** Correlation matrix for the full dataset, with teal indicating positive and blue indicating negative correlations; values exceeding 0.8 highlight potentially collinear predictors that may require attention before modeling

Beyond variable classification, the module performs quality control checks including missing value detection, outlier identification using interquartile range (IQR), and correlation analysis to reveal redundant or collinear factors. Missing values are handled through complete case deletion—any row containing a missing value is removed prior to ML training. Interactive visualizations include distribution charts, outlier boxplots, and correlation heatmaps (Fig. 3A–C). This ensures minimum design requirements (≥1 predictor, ≥1 response variable) are met with proper categorical encoding and complete quality assessment for robust optimization workflows.

#### Build models

The ML module creates predictive models that predict experimental outcomes instantly. Using the "caret" package framework [46], the module processes classified experimental data (Type B, C, and D variables) to build predictive models linking culture conditions to experimental responses. ADAM offers nine distinct algorithms spanning three categories (Table S1): linear methods (PLS, Elastic Net), ensemble methods (RF, XGBoost, ESR), and non-linear methods (MARS, K-NN, SVM, ANN). Users can run all models simultaneously for automated spot-check comparison, select specific models, or run individual algorithms. This diversity ensures high predictive accuracy across varied datasets, as biological relationship complexity is unknown beforehand. The recommended approach is automated spot-check comparison, training all algorithms to identify the best fit. Model selection is purely data-driven: users select the model with highest R^2^ and lowest RMSE on the unseen test set, ensuring the most accurate predictions for optimization.

*Preprocessing pipeline:* Automatic detection of missing values and removal of non-varying variables. Center-scale normalization for Type B quantitative predictors is implemented but currently disabled pending full validation of the required back-transformation steps throughout the optimization and solution evaluation modules. Users working with predictors spanning large concentration ranges—such as plant growth regulators—are advised to prioritize tree-based algorithms (Random Forest, XGBoost) which are scale-invariant, until advanced preprocessing options become available.

**Fig. 4:**
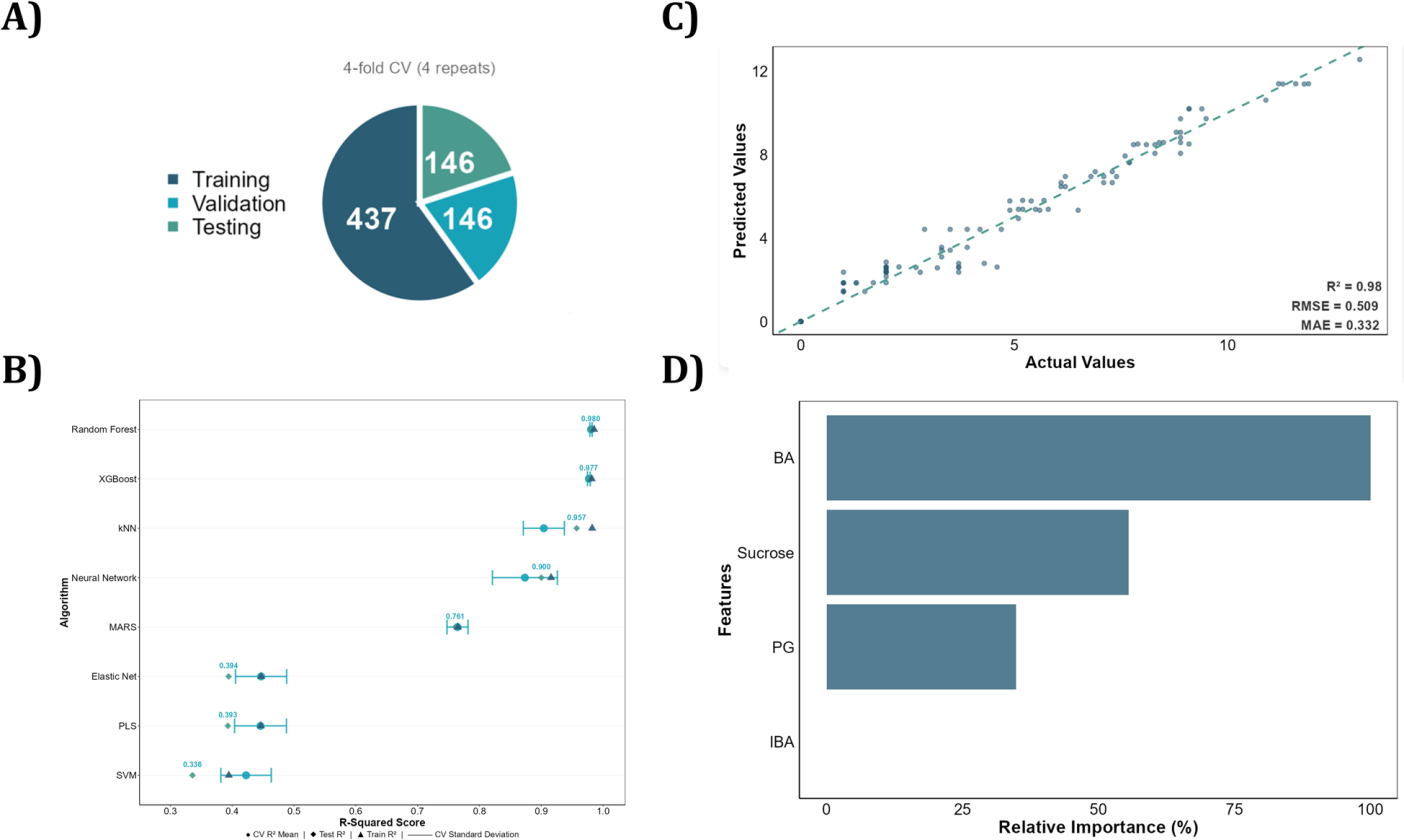
ADAM—Build Models module. **A** Data partitioning visualization showing 75% training and validation and 25% test set split, with fourfold cross-validation repeated 5 times as the training procedure for Use Case I [36]. **B** Model performance comparison across eight ML algorithms (excluding ESR) based on R^2^ scores from cross-validation (CV), training, and test sets for the response variable shoot number (SN). **C** Prediction performance visualization on the test set for the selected RF model, showing strong correlation between predicted and actual values for shoot number (SN). **D** Feature importance analysis revealing the relative contribution of predictor variables to the RF model’s predictive accuracy, with BA demonstrating dominant importance (~100%) for SN prediction

*Data splitting and cross validation:* Automatic splitting into training (60–70%), validation (15–20%), and test (15–20%) sets. Repeated *k*-fold cross-validation (default: fivefold with 3 repetitions) trains and tunes models. For small datasets (<50 observations), Leave-One-Out Cross-Validation (LOOCV) maximizes data utilization (Fig. 4A). The "caret" framework handles hyperparameter optimization through grid search during cross-validation.

*Performance metrics:* Three key metrics quantify model accuracy: coefficient of determination (R^2^) measures the proportion of response variability explained by the model; Root Mean Square Error (RMSE) measures average prediction error; Mean Absolute Error (MAE) provides average absolute prediction error. Cross-validation performance represents mean accuracy across folds during hyperparameter optimization (Fig. 4B, C).

*The coefficient of determination (R*^*2*^*)*: This is the most common metric for assessing model accuracy. It quantifies the proportion of the total response variability in the data that the model can explain, calculated as Eq. (1):

1 $${\mathrm{R}}^{2} = 1 - \frac{{\sum (y_{actual} - y_{predicted} )^{2} }}{{\sum (y_{actual} - \hat{y}_{acutal} )^{2} }}$$

where *y*_*actual*_ are observed response values, *y*_*predicted*_ are model predictions, and $\hat{y}_{acutal}$ is the mean of observed values.

*Root Mean Square Error (RMSE)*: This metric measures the average difference between the model’s predicted values and the actual values, calculated as Eq. (2):

2 $$RMSE=\sqrt{\frac{{\sum ({y}_{actual}-{y}_{predicted})}^{2}}{n}}$$

where *n* denotes the number of response observations in the corresponding set, *y*_*actual*_ the response value and *y*_*predicted*_ the predicted response value.

*Mean Absolute Error (MAE)*: This metric represents the simple average of the absolute errors, providing a clear and direct measure of the average prediction error, calculated as Eq. (3):

3 $$MAE=\frac{\sum {|y}_{actual}-{y}_{predicted}|}{n}$$

*Feature importance analysis:* ADAM automatically quantifies each predictor’s relative contribution to model performance using "caret" package functions. For tree-based models (RF, XGBoost), importance derives from mean decrease in accuracy or impurity; for linear models (PLS, Elastic Net), from standardized regression coefficients or loading weights. Normalized importance scores are visualized as ranked bar plots, identifying which culture factors exert strongest influence on predicted responses (Fig. 4D).

*Model results and reproducibility:* To ensure reproducibility, users can specify a random seed for the ML training module, which controls data splitting into training, validation and test set, guaranteeing identical results across repeated runs with the same configuration. Results are presented through performance comparison tables and visualizations (prediction plots, feature importance rankings, cross-validation stability charts). Comprehensive storage includes algorithm parameters, preprocessing transformations, cross-validation settings, performance metrics, and reproducibility metadata (factorial encodings, scaling parameters, random seeds). The platform automatically highlights the best-performing model by comparing R^2^, RMSE, and MAE across all trained algorithms.

#### Optimization

The Optimization module provides model-driven optimization to identify optimal culture conditions using meta-heuristic approaches (evolutionary algorithms and swarm intelligence) implemented through the "ecr" package framework [14]. ADAM embeds trained ML models as fast approximations of true objective functions, transforming slow lab-based searches into rapid, data-driven computational processes. The module implements four distinct algorithms covering single-objective and multi-objective optimization (Table S2).

*Model preloading and caching*: Dynamic preloading loads trained model objects (including preprocessing transformations) into memory at optimization start, avoiding repeated disk reads during thousands of prediction calls. An internal prediction cache stores recently evaluated parameter sets and fitness scores; when identical candidates are generated in subsequent generations, cached values are retrieved instead of recomputing predictions. This reduces computational overhead and accelerates convergence. A separate random seed can be specified for the EA optimization module, controlling population initialization and ensuring that any optimization run can be reproduced exactly given the same parameter configuration.

*Objective function handling*: For single-objective algorithms (GA, PSO), ML predictions serve directly as fitness scores. For multi-objective algorithms (NSGA-II, SMS-EMOA), ADAM automatically normalizes objectives to prevent larger numerical ranges from dominating the search. Individual fitness values are normalized using minimum and maximum observed values from the ML dataset, direction-corrected (converting minimization to maximization), and summed to compute cumulative fitness ranging from 0 (worst) to n (best), where n is the number of objectives. This scalarized metric assesses overall solution quality throughout Optimization and Evaluate modules. SMS-EMOA hypervolume calculation automatically positions reference points slightly beyond worst observed performance (e.g., minimum values plus 10% buffer for maximization objectives). The normalization is calculated as Eq. (4):

4 $${Fitness}_{normalized}= \frac{{{(Fitness}_{raw}-y}_{\mathrm{min}})}{({{y}_{max}-y}_{min})}$$

where *Fitness*_*raw*_ is the raw predicted value, and *y*_*min*_ and and *y*_*max*_ the minimum and maximum observed values for that objective variable in the ML dataset.

Direction correction is then applied to convert minimization objectives into maximization problems Eq. (5):

5 $${Fitness}_{i,corrected}=\left\{\begin{array}{c}{Fitness}_{i,normalized}, \text{if direction}=\mathrm{maximize}\\ {1-Fitness}_{i,normalized}, \text{if direction}=\mathrm{minimize}\end{array}\right.$$

The cumulative normalized fitness aggregates all corrected objectives Eq. (6):

6 $${Fitness}_{cumulative}=\sum_{i=1}^{n}{Fitness}_{i,corrected}$$

where *n* denotes the number of objectives. This scalarized metric ranges from 0 (worst) to n (best) and is used throughout the Optimization and Evaluate modules to assess overall solution quality.

*Parameter space and feasibility control:* The optimization pipeline handles mixed experimental designs, respecting distinct requirements of quantitative (Type B) and categorical (Type C) factors. Type C predictors maintain discrete integer constraints through automatic rounding. Type B predictors have bounds extracted from ML datasets, with optional extrapolation allowing users to explore factor ranges beyond training data (default: 0% expansion). All bounds adapt automatically to any preprocessing transformations applied during model building, and will adjust accordingly once center-scale normalization is fully validated and enabled in a future version.

*Experimental feasibility and constraints:* Flexible penalty-based constraint handling ensures biologically feasible recommendations. Users define constraint rules (e.g., maximum total salt concentration) with configurable penalty weights before optimization. Supported constraints include individual variable bounds (e.g., auxin ≤ 2 mg L^−1^) and sum of predictor bounds (e.g., total PGR ≤ 5.0 mg L^−1^).

*Visualization of the optimization process:* Optimization progress is visualized by raw fitness convergence (best and mean per generation) for single-objective algorithms or scalarized fitness progression and individual objective evolution plots showing trade-off patterns and convergence for each response variable for multi-objective algorithms.

*Solution filtering and analysis:* Distance-based filtering uses clustering to remove redundant solutions, ensuring diverse, representative optimal recommendations. Sequential filtering for multi-objective optimizations allows step-by-step response prioritization. The module incorporates fitness caching, comprehensive history management, and performance metrics including convergence rates, solution quality, computational efficiency, and solution diversity.

#### Evaluate

The fifth and final module of ADAM evaluates the optimization results by comparing the original (uploaded) experimental data with the optimized solutions. This comparison is performed for each selected objective variable, assessing differences in the distributions, mean values, and best observed values between the original and optimized datasets. Results are visualized using violin plots. For multi-objective optimizations, ADAM calculates the cumulative normalized fitness (Fitness_cumulative_) for both the original dataset and optimized solutions using the same normalization and direction correction procedure. The results are presented in tabular form, providing a quantitative measure of the overall improvement achieved across all objectives. Higher cumulative fitness values indicate superior overall performance.

### Software implementation, hosting and performance

To make these advanced methods accessible for the commercial biotechnology sector and academia, the ADAM AI platform v2.1.6 was developed as a browser-based application [https://adam.quanta-biolabs.com]. The platform is currently deployed as a virtual machine in a cloud environment, running Ubuntu 24.04 with 4 CPU cores and 16 GB RAM. ADAM v2.1.6 currently supports up to 10 concurrent user sessions through a 10-worker configuration; however, this setup can be easily scaled to accommodate higher user loads as needed. This cloud-based setup ensures global accessibility via any standard web browser (Chrome tested), eliminating dependencies on local computational resources, high-performance hardware, or proprietary software licenses for end-users.

## Validation and performance

### Use Case I: Multi-objective optimization of medium composition for *chrysanthemum* shoot proliferation

To demonstrate ADAM’s pure multi-objective optimization capabilities, we replicated and extended the study of Hesami et al. [36] on *Chrysanthemum* (*Dendranthema* × *grandiflorum* Ramat.) shoot regeneration. This case showcases Pareto-based NSGA-II optimization for inherently conflicting objectives—maximizing shoot proliferation parameters while minimizing basal callus formation.

#### Database

We validated ADAM using the chrysanthemum (*Dendranthema* × *grandiflorum* Ramat.) shoot regeneration dataset from Hesami et al. [36], comprising 81 treatment combinations (34 factorial design, 729 observations) with 4 input variables (BAP, IBA, phloroglucinol, and sucrose) and 4 response variables: proliferation rate (PR, %), shoot number (SN), shoot length (SL, cm), and basal callus weight (BCW, g). The original study employed RBF-NSGA-II for multi-objective optimization, simultaneously maximizing PR, SN, and SL while minimizing BCW to address the conflicting objectives between shoot proliferation and undesirable callus formation. Their optimized medium (2.16 µM BAP, 0.14 µM IBA, 0.29 mM phloroglucinol, 87.63 mM sucrose) was experimentally validated, achieving 100% PR (predicted: 98.85%), 12.87 shoots per explant (predicted: 13.32), 4.63 cm SL (predicted: 4.83 cm), and 0.06 g BCW (predicted: 0.08 g).

#### ML training

The ML training module of ADAM processed the dataset using eight different algorithms (excluding ESR) in spot-check mode with fivefold cross-validation repeated 5 times (Random seed: 126), following the original study’s approach. The data was split into 75% training (547 observations) and 25% testing (182 observations) sets. Table 2 presents the top three performing models for each response variable based on Test R^2^ values.

**Table 2:** ADAM´s performance metrics of the top three ML models for each response variable in Use Case I from Hesami et al. [36]

Algorithm	Response variable		CV R^2^	Train R^2^	Test R^2^	CV RMSE	Train RMSE		Test RMSE	Test MAE	
XGBoost	PR	**0.92 ± 0.01**		0.93	**0.91**	**10.65 ± 0.75**		**9.84**	**10.99**	**7.79**	
RF	PR	**0.92 ± 0.01**		**0.94**	**0.91**	**10.65 ± 0.70**		9.65	11.06	7.95	
kNN	PR	0.87 ± 0.02		**0.94**	0.88	13.87 ± 1.30		9.53	12.64	8.32	
RF	SN	**0.98 ± 0.00**		**0.99**	**0.98**	**0.51 ± 0.04**		0.44	**0.52**	**0.35**	
kNN	SN	0.90 ± 0.01		**0.99**	**0.98**	1.22 ± 0.24		**0.43**	0.53	0.36	
XGBoost	SN	**0.98 ± 0.00**		0.98	0.97	0.57 ± 0.05		0.49	0.56	0.42	
kNN	SL	0.96 ± 0.01		**0.98**	**0.97**	0.44 ± 0.04		**0.33**	**0.37**	**0.26**	
RF	SL	**0.97 ± 0.01**		**0.98**	**0.97**	0.38 ± 0.03		**0.33**	0.38	**0.26**	
XGBoost	SL	**0.97 ± 0.01**		0.97	**0.97**	**0.37 ± 0.03**		0.34	0.38	0.28	
XGBoost	BCW	**0.66 ± 0.05**		0.69	**0.64**	**0.06 ± 0.00**		**0.06**	**0.07**	**0.04**	
RF	BCW	0.65 ± 0.06		**0.71**	0.62	0.07 ± 0.01		**0.06**	**0.07**	**0.04**	
NNet	BCW	0.59 ± 0.04		0.61	0.57	0.07 ± 0.01		0.07	**0.07**	0.05	

Models were ranked by Test R^2^ and evaluated using cross-validation (CV), training, and test set performance

Bold values indicate the best performance in each column

#### Optimization

**Fig. 5:**
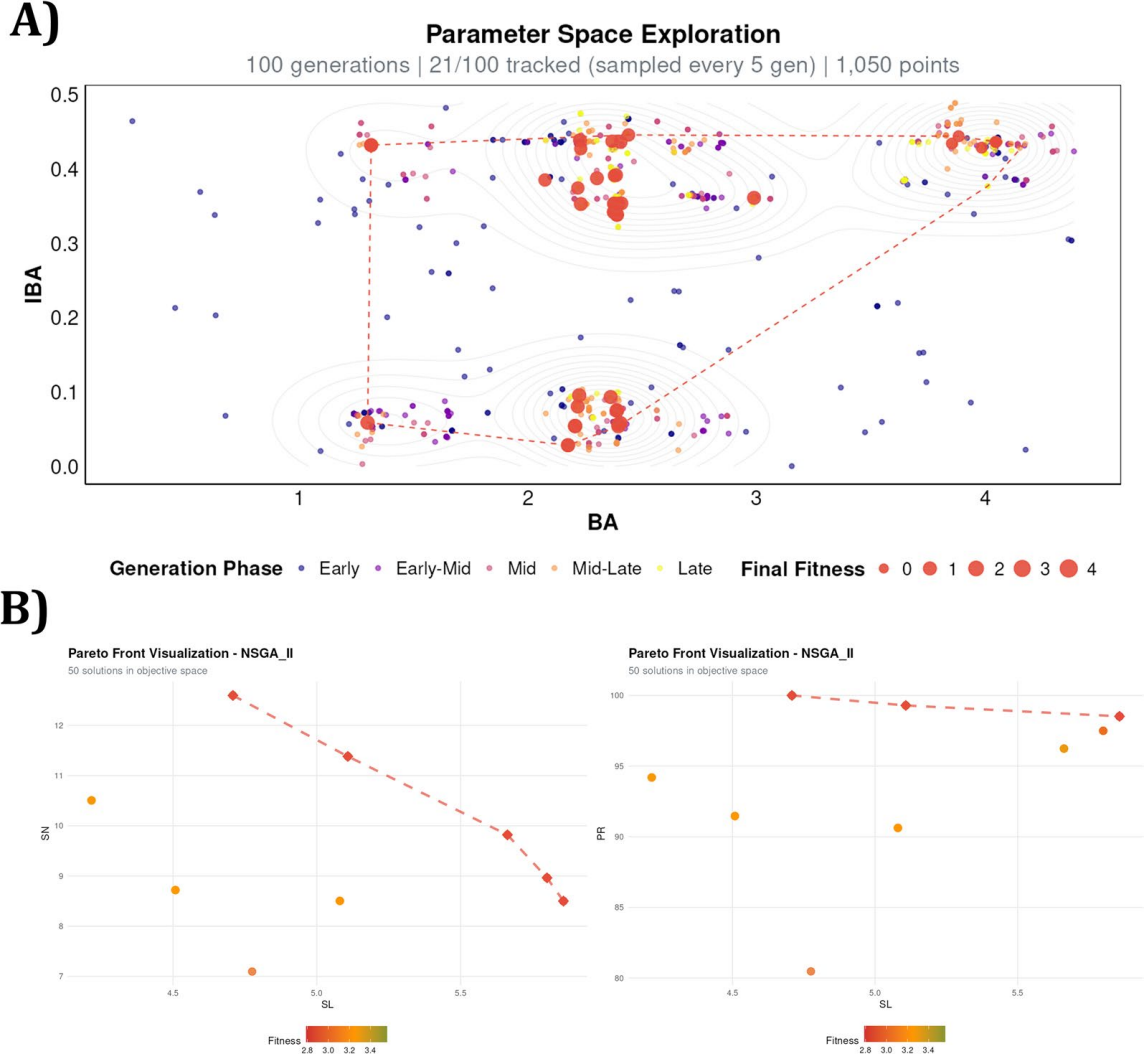
ADAM optimization visualizations for Use Case I. **A** Parameter Space Exploration plot showing the trajectory of NSGA-II across 100 generations in the two-dimensional space of the key growth regulators BA (6-benzylaminopurine) and IBA (indole-3-butyric acid). Each point represents an evaluated candidate solution, colored by generation phase (Early to Late) and sized by cumulative normalized fitness, illustrating how the algorithm progressively focuses on high-fitness regions while maintaining population diversity. Contour lines represent the estimated fitness landscape derived from the surrogate model. The *dashed rectangle* and connecting lines highlight the zoom region showing solution clustering in the optimal BA/IBA range. **B** Pareto Front Visualizations showing the 50 non-dominated solutions identified by NSGA-II in objective space, plotting shoot number (SN) and proliferation rate (PR) against shoot length (SL). Point color indicates cumulative normalized fitness. The *dashed line* connects Pareto-optimal solutions, illustrating the trade-off between competing objectives and enabling informed selection of biologically meaningful candidate media formulations

ADAM’s multi-objective optimization approach was applied to simultaneously optimize all four response variables, identifying non-dominated solutions solutions that balance competing objectives (Table 3). The NSGA-II algorithm was configured with a population size of 50 individuals and 20 offspring per generation, running for 100 generations to accumulate 2,050 total fitness evaluations. The evolutionary operators were set with a mutation probability of 20% and crossover probability of 70%. Simulated Binary Crossover (SBX) was employed with a distribution index (η) of 15, while polynomial mutation used η = 25 to control the exploration–exploitation balance during the search process (Random seed: 121). The parameter space exploration and resulting Pareto front are visualized in Fig. 5A, B.

**Table 3:** Comparison of optimized medium compositions for chrysanthemum shoot proliferation

Type	BAP (µM)	IBA (µM)	PG (µM)	Sucrose (mM)		Predicted PR (%)	Predicted SN (count)	Predicted SL (cm)	Predicted BCW (g)	
Hesami et al. [36]	2.16	0.14	0.29	87.63		98.85	13.32	4.83	0.08	
ADAM—NSGAII	2.91	0.35	**0.22**	101.67		**100.00**	**12.60**	4.71	**0.05**	
ADAM—NSGAII	2.12	0.21	0.29	111.07		**100.00**	**12.60**	4.71	**0.05**	
ADAM—NSGAII	3.10	0.41	0.39	95.77		**100.00**	**12.60**	4.71	**0.05**	
ADAM—NSGAII	3.10	0.21	0.28	112.91		99.29	11.38	5.11	0.06	
ADAM—NSGAII	2.26	**0.08**	0.38	81.27		99.29	11.38	5.11	0.06	
ADAM—NSGAII	2.93	0.10	0.39	113.76		94.20	10.51	4.22	**0.05**	
ADAM—NSGAII	2.25	0.39	0.30	98.76		94.20	10.51	4.22	**0.05**	
ADAM—NSGAII	3.58	0.42	0.29	**69.66**		94.20	10.51	4.22	**0.05**	
ADAM—NSGAII	3.24	0.45	0.29	99.07		96.24	9.82	**5.66**	0.13	
ADAM—NSGAII	**2.00**	0.38	0.40	83.44		96.24	9.82	**5.66**	0.13	

The table shows the original solution from Hesami et al. [36] and optimized candidate solutions identified by ADAM

Bold values indicate top performance for each predicted output variable and minimum concentration for each input variable (BAP, IBA, PG, Sucrose)

#### Discussion

ADAM’s ensemble methods (XGBoost, RF) achieved comparable or superior performance to the original RBF approach across most response variables. For PR, ADAM achieved Test R^2^ of 0.91 (vs. 0.88 original) with improved RMSE (10.99 vs. 13.38). SN prediction showed substantial improvement (Test R^2^ = 0.98 vs. 0.91; RMSE = 0.52 vs. 1.20, representing 56% error reduction). SL performance was equivalent, while BCW prediction remained challenging for both approaches (ADAM R^2^ = 0.64 vs. 0.76 original), though absolute prediction errors were identical (RMSE = 0.07 g), reflecting the high biological variability in callus formation. ADAM’s automated algorithm selection produced models matching or exceeding manually optimized RBF performance while requiring minimal user expertise.

Multi-objective optimization identified candidate optimized solutions spanning 94.2–100% PR, 9.82–12.60 shoots per explant, 4.22–5.66 cm SL, and 0.05–0.13 g BCW. ADAM’s top predictions matched the laboratory-validated results from Hesami et al. [36], with 100% PR and BCW within 0.01 g of experimental values. The comprehensive factorial design (729 observations) ensured optimal regions were well-sampled, enabling accurate model predictions without extrapolation. ADAM’s primary contribution was automated identification and ranking of alternative optimal protocols from the complete dataset, eliminating manual screening and providing quantitative guidance for protocol selection based on objective priorities.

ADAM successfully identified the optimal parameter combinations that were already present in the experimental design, demonstrating its capability to extract and rank the best solutions from comprehensive datasets. The parameter space exploration revealed multiple high-fitness regions in the BA/IBA space (Fig. 5A), indicating that distinct growth regulator combinations can achieve comparable performance—a finding that would not be apparent from single-point optimization approaches. The Pareto front visualization (Fig. 5B) further illustrates the inherent trade-off between competing objectives, such as the antagonistic relationship between shoot number and shoot length, providing researchers with a transparent basis for protocol selection according to their specific experimental priorities.

### Use Case II: Multi-objective optimization of medium composition for pistachio micropropagation using NSGA-II and SMS-EMOA algorithms

To demonstrate ADAM’s multi-objective Pareto optimization with constraint handling, we applied the platform to data for *Pistacia vera* cv. ‘Ghazvini’ rootstock based on Nezami-Alanagh et al. [57]. This case introduces minimum performance constraints for all objectives while comparing ADAM’s NSGA-II approach, which generates diverse trade-off solutions within constraint boundaries. This approach was compared to the original study’s weighted single-composite optimization that reduced a multi-objective task to a single-objective problem.

#### Database

We validated ADAM using the pistachio (*Pistacia vera* cv. ‘Ghazvini’) micropropagation dataset from Nezami-Alanagh et al. [57], comprising 58 treatment combinations testing mineral nutrition (three basal media: MS [52], DKW [18], and GNH [28]), vitamins (thiamine-HCl, nicotinic acid, pyridoxine–HCl), glycine, and plant growth regulators (BAP, IBA). The dataset included 25 input variables and four response variables: propagation factor (PF), shoot length (SL), total fresh weight (TFW), and healthy fresh weight (HFW).

The original study employed ANN-GA with single-composite optimization (weighted objectives: PF = 10, SL = 9, HFW = 8, TFW = 7) and performance constraints (PF > 4, SL > 20 mm, TFW and HFW > 0.5 g) to develop Pistachio Optimal Medium (POM). Their ANN models achieved high predictability: PF (Train R^2^ = 0.95, Test R^2^ = 0.82), SL (Train R^2^ = 0.94, Test R^2^ = 0.74), TFW (Train R^2^ = 0.93, Test R^2^ = 0.71), and HFW (Train R^2^ = 0.86, Test R^2^ = 0.81). Laboratory validation confirmed POM’s superior performance (3.73 shoots per explant, 23.3 mm SL, 0.45 g TFW, 0.26 g HFW) compared to standard media controls.

#### ML training

The ML training module of ADAM processed the dataset using eight different algorithms (excluding ESR) in spot-check mode with fourfold cross-validation repeated 8 times (Random seed: 117), following the original study’s approach. The data was split into 75% training (44 observations) and 25% testing (14 observations) sets. Table 4 presents the top three performing models for each response variable based on Test R^2^ values. The four response variables (PF, SL, TFW, and HFW) were all modeled with high predictive accuracy, as reflected by the strong R^2^ values and low prediction errors (Table 4).

**Table 4:** ADAM´s performance metrics of the top three ML models for each response variable in Use Case II (*Pistacia vera* shoot proliferation) from Nezami-Alanagh et al. [57]

Algorithm	Response variable	CV R^2^	Train R^2^	Test R^2^	CV RMSE	Train RMSE	Test RMSE	Test MAE
XGBoost	**PF**	**0.88 ± 0.07**	0.93	**0.89**	**0.51 ± 0.11**	0.37	**0.50**	**0.38**
Random Forest	PF	0.86 ± 0.08	**0.96**	0.88	0.52 ± 0.13	**0.26**	0.51	0.43
MARS	PF	0.82 ± 0.10	0.93	0.82	0.60 ± 0.12	0.35	0.62	0.45
Random Forest	SL	**0.80 ± 0.10**	**0.94**	**0.86**	**3.36 ± 0.81**	**1.67**	**2.67**	2.67
MARS	SL	0.72 ± 0.14	0.81	**0.86**	3.97 ± 1.01	3.01	2.73	2.45
XGBoost	SL	0.75 ± 0.12	0.88	0.84	3.64 ± 0.95	2.39	2.87	**2.35**
Random Forest	TFW	0.84 ± 0.07	**0.94**	**0.92**	0.15 ± 0.02	**0.08**	**0.09**	**0.07**
XGBoost	TFW	**0.85 ± 0.07**	0.90	**0.92**	**0.14 ± 0.03**	0.10	**0. 09**	0.08
MARS	TFW	**0.85 ± 0.08**	0.88	0.89	**0.14 ± 0.03**	0.12	0.11	0.09
MARS	HFW	0.69 ± 0.15	0.70	**0.57**	0.09 ± 0.02	0.09	**0.10**	0.08
Random forest	HFW	**0.79 ± 0.10**	**0.91**	0.56	**0.07 ± 0.01**	**0.05**	**0.10**	**0.07**
XGBoost	HFW	**0.79 ± 0.10**	0.88	0.47	**0.07 ± 0.01**	**0.05**	0.11	0.08

Models were ranked by Test R^2^ and evaluated using cross-validation (CV), training, and test set performance

Bold values indicate the best performance in each column

Regarding PF, the XGBoost model yielded the highest Test R^2^ (0.89) and maintained elevated training performance (Train R^2^ = 0.93). Prediction of SL showed strong reliability across top models, with Random Forest achieving the best Test R^2^ (0.86), followed closely by MARS (Test R^2^ = 0.86). For TFW, three algorithms performed robustly, with both Random Forest and XGBoost achieving the highest Test R^2^ (0.92), though Random Forest showed slightly lower test error (Test RMSE = 0.09; MAE = 0.07). Finally, regarding HFW, all models showed moderate predictive strength, with MARS delivering the best Test R^2^ (0.57), although overall performance was lower than for other response variables, with test errors ranging from RMSE = 0.10–0.11 and MAE = 0.07–0.08.

#### Optimization

ADAM’s multi-objective optimization approach was applied to simultaneously optimize all four response variables, identifying non-dominated solutions that balance competing objectives (Table 5). Two complementary evolutionary algorithms were employed to ensure robust identification of optimal media formulations: NSGA-II (Non-dominated Sorting Genetic Algorithm II) and SMS-EMOA (S-Metric Selection Evolutionary Multi-Objective Algorithm).

**Table 5:** Comparison of optimized medium compositions for pistachio shoot proliferation

Component	DKW	MS	GNH	POM	This study I NSGA II*	This study II SMS–EMOA*	
Macronutrients							
KNO_3_	–	1900	25.0	558	496.5	42.1	
NH_4_NO_3_	1417	1650	1650	1532	1577.0	1607.2	
Ca(NO_3_)_2_·4H_2_O	1960	–	800	555	1004.1	1163.9	
CaCl_2_·2H_2_O	147	440	0.0	35	12.1	331.4	
MgSO_4_·7H_2_O	740	370	540	468	566.1	616.8	
KH_2_PO_4_	229	170	300	223	291.7	291.8	
K_2_SO_4_	1559	–	–	321	566.1	389.6	
NaH_2_PO_4_·H_2_O	–	–	50.0	24	2.7	40.0	
Micronutrients							
MnSO_4_·4H_2_O	44.6	22.3	22.3	41.5	26.8	30.8	
ZnSO_4_·7H_2_O	16.4	8.6	8.6	10.2	11.3	9.7	
H_3_BO_3_	4.8	6.2	6.2	4.8	6.1	5.6	
KI	–	0.8	0.8	0.8	0.6	0.5	
CuSO_4_·5H_2_O	0.25	0.025	0.025	0.11	0.13	0.14	
Na_2_MoO_4_·2H_2_O	0.39	0.25	0.25	0.30	0.33	0.25	
CoCl_2_·6H_2_O	0.0	0.025	0.025	0.02	0.02	0.01	
FeSO_4_·7H_2_O	33.4	27.9	27.9	31.2	30.5	28.1	
Na_2_·EDTA·2H_2_O	44.7	37.3	37.3	41.71	37.64	38.79	
Vitamins							
Thiamine-HCl	2.0	0.1	0.1	5.3	7.5	2.9	
Nicotinic-acid	1.0	0.5	0.5	0.7	0.09	0.10	
Pyridoxine–HCl	–	0.5	0.5	0.6	0.02	0.18	
Glycine	2.0	2.0	2.0	0.25	0.88	0.22	
Plant growth regulators							
IBA	0.1	0.1	0.1	0.09	0.03	0.001	
BAP	0.5	0.5	0.5	1.5	1.58	1.68	
Response variables							
PF	2.4 ± 0.3	1.8 ± 0.1	3.2 ± 0.3	4.4	4.6	4.0	
SL (mm)	17.4 ± 1.6	24.7 ± 1.3	18.9 ± 2.8	28.7	21.2	28.9	
TFW (g)	0.4 ± 0.02	0.4 ± 0.05	0.47 ± 0.08	1.1	1.1	1.1	
HFW (g)	0.2 ± 0.01	0.2 ± 0.03	0.27 ± 0.04	0.5	0.5	0.5	

The table shows the original POM solution from Nezami-Alanagh et al. [57] and optimized candidate solutions identified by ADAM using NSGA-II and SMS-EMOA. All concentration values in mg L^−1^

* Only solution with highest cumulative Fitness_normalized_ is depicted

*Model integration*: Both algorithms optimized the same four objectives using the selected machine learning models: XGBoost for PF (Test R^2^ = 0.89) and RF for SL (R^2^ = 0.86), RF for TFW (R^2^ = 0.82), and MARS for HFW (R^2^ = 0.57).

*NSGA-II configuration: *The NSGA-II algorithm was configured with a population size of 50 individuals and 50 offspring per generation, running 150 generations to accumulate 7,550 total fitness evaluations. The evolutionary operators were set with a mutation probability of 20% and crossover probability of 70%. Simulated Binary Crossover (SBX) was employed with a distribution index (η) of 15, while polynomial mutation used η = 25 to control the exploration–exploitation balance during the search process (Random seed: 121).

*SMS-EMOA configuration*: To complement NSGA-II’s diversity-focused approach, SMS-EMOA was implemented with a steady-state strategy using a population size (μ) of 100 individuals and generating 1 offspring (λ) per iteration. The algorithm ran for 2000 generations to accumulate 2100 total fitness evaluations. SMS-EMOA employs hypervolume contribution as the selection criterion, which provides superior convergence properties for problems with more than two objectives (Random seed: 121).

#### Discussion

ADAM demonstrated superior or equivalent predictive accuracy across all growth parameters compared to the ANN-GA approach of Nezami-Alanagh et al. [57]. ADAM achieved Test R^2^ of 0.89 for PF (vs. 0.82 original), 0.92 for TFW (vs. 0.71), and 0.86 for SL (vs. 0.74), though HFW prediction remained challenging (R^2^ = 0.57).

Multi-objective optimization using NSGA-II and SMS-EMOA generated formulations with predicted performance comparable to the original POM. NSGA-II predicted 4.6 shoots per explant, 21.2 mm SL, 1.1 g TFW, and 0.5 g HFW; SMS-EMOA predicted 4.0 shoots, 28.9 mm, 1.1 g, and 0.5 g, respectively, compared to the original POM values of 4.4 shoots, 28.7 mm, 1.1 g, and 0.5 g.

The substantial compositional differences between algorithms (e.g., KNO₃ ranging from 42 to 496 mg L^−1^) despite similar predicted performance suggest either multiple biologically equivalent pathways to optimal growth or model prediction uncertainty within the explored parameter space. This highlights the importance of experimental validation and demonstrates that ADAM’s multi-algorithm approach can reveal alternative formulations, which merit experimental validation to assess biological equivalence and potential cost-effectiveness.

It should be noted that Use Case II is based on a relatively small dataset (58 observations, 25 input variables), which imposes inherent limitations on model generalizability. The consistency between CV R^2^ and Test R^2^ values across response variables suggests that overfitting was not substantial; however, results from small datasets should be interpreted with caution and larger validation sets would strengthen confidence in the reported findings.

## General discussion

The development and validation of ADAM directly addressed the three critical barriers identified in our literature analysis (Table 1; Fig. 1): reliance on licensed software, limited adoption of multi-objective optimization, and lack of integrated, accessible and reproducible workflows. Through two complementary validation studies and mathematical benchmarking (Appendix S3, ZDT2 function), we demonstrated that ADAM provides research-grade optimization capabilities while eliminating traditional barriers to entry. Table 6 directly compares ADAM against established computational platforms, demonstrating that ADAM uniquely combines open access, complete workflow integration (DoE → ML → Optimization), automated model selection, and true multi-objective optimization—features that are either absent or require expensive licenses in existing platforms.

**Table 6:** Comparison of computational platforms integrating experimental design, predictive modeling, and optimization for biotechnological processes

Feature	ADAM	JMP Pro	Minitab	Design-Expert	INForm®	SUMO Toolbox	FormRules
Interface	Guided browser-based	Desktop	Desktop	Desktop	Desktop	MATLAB	Desktop
Access and cost	Free	Paid	Paid	Paid	Paid	MATLAB-licensed	Paid
Complete workflow	Yes	Yes	Partial	Partial	Partial	Yes	No
DoE types	5	>9	>10	>7	–	6	–
ML algorithms	9	~20	0 (RSM only)	0 (RSM only)	ANN	> 10	1 (Neurofuzzy)
Automated spot-check	Yes	Yes	No	No	No	Yes	No
Multi-objective optimization	Yes	Single-composite	Single-composite	Single-composite	Single-composite	Yes	No
Optimization algorithms	4	Desirability/RSM	Desirability/RSM	Desirability/RSM	GA	> 8	0

Complete workflow indicates whether the platform provides integrated DoE design, predictive modeling, and optimization in a unified environment. "Partial" indicates platforms that support some but not all workflow stages

A significant challenge in current literature is the lack of methodological transparency and standardization. Many studies do not report critical details such as data partitioning strategies, cross-validation procedures, or specific performance metric formulations, making model reliability challenging to assess and results difficult to compare. ADAM addresses these limitations through enforced best practices: automatic train-validation-test splitting, mandatory cross-validation with adaptive selection (k-fold for larger datasets, LOOCV for small datasets < 50 observations), and standardized reporting of multiple performance metrics (R^2^, RMSE, MAE). This built-in rigor ensures quality standards and provides a framework for harmonizing optimization pipelines across the plant tissue culture community.

Our validation studies demonstrate that ADAM’s automated ML modeling achieved predictive performance comparable to or exceeding manually optimized approaches. Models achieved R^2^ values of 0.91–0.98 for Use Case I and >0.80 for most responses in Use Case II, despite challenging dataset characteristics (n = 58, 25 input variables).

A key innovation is ADAM’s seamless integration of multi-objective optimization through NSGA-II and SMS-EMOA algorithms. Traditional single-objective approaches simplify multi-objective problems using weighted composite functions, requiring arbitrary priority assignment and obscuring trade-offs. ADAM’s Pareto-based approach generates diverse non-dominated solutions, enabling informed decisions based on specific priorities and constraints. ADAM’s value extends beyond discovering novel solutions by systematically identifying and ranking optimal protocols from large experimental datasets, eliminating manual screening and providing quantitative selection guidance. Both use cases demonstrated this capability by identifying multiple optimal culture conditions balancing competing objectives.

While ADAM was tested here and designed for plant tissue culture optimization, the underlying ML-EA framework is applicable to any system involving expensive experimental evaluations and complex factor interactions. Potential applications include biopharmaceutical formulation design (optimizing excipient combinations for protein stability), bioprocess optimization (culture media for microbial or mammalian cell production), controlled environment agriculture (climate chamber or greenhouse parameter optimization), and hydroponic solution formulation. The platform’s modular architecture allows adaptation to diverse biological and biotechnological optimization problems without modification to core algorithms. Domain-independent validation of the pipeline is provided in Appendix S3, where ADAM is applied to the ZDT2 mathematical benchmark function—a well-established multi-objective test problem with a known theoretical Pareto front—confirming that the ML training and optimization modules function correctly independently of any biological context. Applicability to other experimental domains beyond plant tissue culture remains an explicit avenue for future validation.

Practical considerations include experimental design adequacy (see [62]) and potential preprocessing requirements for noisy biological data. For small datasets (<50 observations), careful test set selection is critical, and users should recognize that model generalization may be limited when training data inadequately represents the parameter space. While ADAM predicts responses within or near the training data distribution, extrapolation beyond observed ranges should be approached cautiously, and predictions should be validated experimentally using multiple genotypes. The exploration–exploitation tradeoff inherent to every optimization campaign deserves explicit consideration, particularly in biological applications where experiments are slow and expensive. In ADAM’s current retrospective framework, broad exploration is promoted during data collection through space-filling DoE designs, while exploitation is handled by the EA during surrogate optimization. Sequential design strategies from related fields—including Bayesian experimental design in pharmaceutical formulation and response surface methodology in bioprocess optimization—highlight avenues where ADAM’s current capabilities could be extended and systematically benchmarked in future work.

Current limitations include absence of uncertainty quantification for predictions and lack of active learning or sequential experimental design strategies for iterative protocol refinement. Future enhancements will address these limitations and expand functionality through Bayesian optimization, sensitivity analysis, robustness validation, active learning modules, additional ML algorithms, expanded constraint handling, and pair/corner plot visualizations for surrogate landscape exploration.

ADAM operates entirely in-memory without permanent data storage, ensuring proprietary culture media recipes remain under user control—critical for intellectual property protection. While our validation focused on plant tissue culture applications, broader testing across diverse biotechnological systems and larger user communities will be essential to fully characterize ADAM’s performance across varied experimental contexts and data characteristics.

The ADAM platform represents a step toward democratizing advanced AI-driven optimization in plant biotechnology and related fields. By providing free access to state-of-the-art methods through an intuitive web interface, ADAM enables researchers and companies to apply sophisticated optimization techniques without specialized programming expertise or expensive software licenses. As the platform evolves through community engagement, we anticipate it will contribute to faster development of optimized tissue culture protocols and broader adoption of data-driven approaches across biotechnology.

## Conclusions

Plant tissue culture optimization has been hindered by reliance on trial-and-error experimentation and limited access to advanced computational methods, particularly in resource-constrained settings where specialized software and expertise are unavailable. ADAM addresses these barriers through an open-access, web-based platform that integrates the full ML-EA workflow, from experimental design through multi-objective optimization, using an intuitive interface requiring no programming expertise.

Validation across plant tissue culture applications demonstrated predictive performance equivalent to manually optimized approaches while successfully identifying non-dominated candidate solutions balancing competing objectives.

As plant biotechnology faces increasing demands for rapid protocol development, ADAM represents a step toward transitioning plant culture media design from empirical intuition to a systematic, data-driven approach. By accelerating identification of optimal conditions while reducing experimental burden, the platform enables researchers to focus on biological discovery rather than exhaustive screening. Beyond plant tissue culture, ADAM’s modular architecture enables application to diverse plant biotechnological processes—including transformation protocols, bioreactor optimization, secondary metabolite production, and acclimatization strategies—accelerating translation of computational methods into routine laboratory practice.

## Supplementary Information

Additional file 1: Table S1. Machine learning algorithms in ADAM. ADAM offers three different modeling categories and nine algorithms varying in architectural complexity. Given is also a brief explanation of the advantages of each, the corresponding caret method identifier, and the hyperparameter search grid used during cross-validation. Table S2. Optimization algorithms in ADAM. ADAM offers four optimization algorithms and a brief explanation of the type of optimization, the use and key features when applied in plant tissue culture. Default parameter values are extracted from the ADAM implementation and represent the starting configuration presented to users, all of which can be adjusted prior to running the optimization. Appendix S3. Pipeline validation: ML model training and optimization benchmarking using ZDT2 test function. Comprehensive validation of single-objective (GA) and multi-objective (NSGA-II) optimization performance under realistic conditions with 10% Gaussian noise. Includes synthetic dataset generation (750 samples, 10 variables), ML training results (Table S3.1), single-objective GA optimization results (Figure S3.2), and multi-objective NSGA-II Pareto front analysis (Figure S3.3)

Additional file 2: Data S4. Raw experimental data of Use Case I. Complete dataset from Hesami et al. [36] containing 729 observations from 81 treatment combinations with 4 input variables (BAP, IBA, phloroglucinol, and sucrose concentrations) and 4 response variables (proliferation rate, shoot number, shoot length, and basal callus weight).

## Data Availability

The ADAM web application (v2.1.6) is freely accessible at https://adam.quanta-biolabs.com for academic and commercial use without licensing fees. The source code is available to reviewers and editors during the peer review process via private repository access and will be made available to the research community upon reasonable request following publication. An exemplary dataset is provided in Data S4.

## Abbreviations

AL: Active learning
ANFIS: Adaptive neuro-fuzzy inference system
ANN: Artificial neural network
BAP: 6-Benzylaminopurine
BBD: Box-Behnken design
BBO: Biogeography-based optimization
CCD: Central composite design
CHAID: Chi-square automatic interaction detection
CV: Cross validation
DoE: Design of experiments
DKW: Driver and Kuniyuki Walnut medium
ENet: Elastic net
ESR: Ensemble stacking regression
EAs: Evolutionary algorithms
FFD: Full factorial design
FOA: Fruit fly optimization algorithm
GA: Genetic algorithm
GEP: Gene expression programming
GNH: Garoosi, Nezami and Haddad
GP: Gaussian process
HMM: Hidden Markov model
IBA: Indole-3-butyric acid
IQR: Interquartile range
ISA: Interior search algorithm
KNN: K-nearest neighbors
LHS: Latin hypercube sampling LHS
MAE: Mean absolute error
MARS: Multivariate adaptive regression splines
ML: Machine learning
MLR: Multiple linear regression
MRA: Multiple regression analysis
MS: Murashige and Skoog
NAA: 1-Naphthaleneacetic acid
NSGA-II: Non-dominated sorting genetic algorithm II
PGRs: Plant growth regulators
PLS: Partial least squares
PSO: Particle swarm optimization
POM: Pistachio optimized medium
RD: Random design
RF: Random forest
RMSE: Root mean square error
RSM: Response surface methodology
SMS-EMOA: S-Metric Selection Evolutionary Multi-Objective Algorithm
SOS: Symbiotic organisms search
SVR: Support vector regression
XGBoost: Extreme gradient boosting
