# Supplementary material for: ADAM: advanced design and AI-driven modeling for plant tissue culture media optimization
Source: Plant Methods. 2026 Apr 26;22:44. doi: 10.1186/s13007-026-01534-5 (PMC13130802; doi:10.1186/s13007-026-01534-5)
Supplement: Supplementary file 1 — Additional file 1: Table S1. Machine learning algorithms in ADAM. ADAM offers three different modeling categories and nine algorithms varying in architectural complexity. Given is also a brief explanation of the advantages of each, the corresponding caret method identifier, and the hyperparameter search grid used during cross-validation. Table S2. Optimization algorithms in ADAM. ADAM offers four optimization algorithms and a brief explanation of the type of optimization, the use and key features when applied in plant tissue culture. Default parameter values are extracted from the ADAM implementation and represent the starting configuration presented to users, all of which can be adjusted prior to running the optimization. Appendix S3. Pipeline validation: ML model training and optimization benchmarking using ZDT2 test function. Comprehensive validation of single-objective (GA) and multi-objective (NSGA-II) optimization performance under realistic conditions with 10% Gaussian noise. Includes synthetic dataset generation (750 samples, 10 variables), ML training results (Table S3.1), single-objective GA optimization results (Figure S3.2), and multi-objective NSGA-II Pareto front analysis (Figure S3.3) [file 13007_2026_1534_MOESM1_ESM.docx]

**Table S1 – Machine learning algorithms in ADAM**

***Table S1:*** *ADAM offers three different modeling categories and nine algorithms varying in architectural complexity. Given is also a brief explanation of the advantages of each, the corresponding caret method identifier, and the hyperparameter search grid used during cross-validation.*

| **Category** | **Model** | **Description  (User benefits)** | **Caret method** | **Key hyperparameters & search grid** |
| --- | --- | --- | --- | --- |
| **Linear methods** | Partial Least Squares (PLS) | Finds simple, underlying patterns (latent variables) that maximize the connection between the *inputs* and the measured outcomes | pls | ncomp: {1, 2, 3} |
|  | Elastic Net (Enet) | A robust, regularized regression method that simultaneously selects the most important factors and reduces the influence of less important factors | glmnet | alpha: {0, 0.1, …, 1.0}; lambda: auto path (alpha=0 → Ridge; alpha=1 → Lasso) |
| **Ensemble methods** | Random Forest (RF) | Creates hundreds of simple decision trees and averages their predictions, which makes the result very reliable and stable | rf | mtry: {floor(√p), floor(√p /2), floor(√p ×2)}; ntree fixed at 500 |
|  | XGBoost | A powerful method that builds a sequence of decision trees, where each new tree corrects the errors made by the previous ones, leading to highly accurate results | xgbTree | nrounds: {50,100,150}; max_depth: {1,2,3}; eta: {0.3,0.4}; gamma: 0; colsample_bytree: {0.6,0.8}; min_child_weight: 1; subsample: {0.5,0.75,1.0} |
|  | Ensemble Stacking Regression (ESR) | A highly advanced method that combines the predictions of several individual models into a final, superior prediction using a second level "meta-learner." | custom stacking | Inherits grids of base learners |
| **Non-Linear methods** | Multivariable Adaptative Regression Splines (MARS) | Creates a flexible, non-parametric model by building small, interconnected linear segments ("splines") that adapt well to sharp changes in plant response | earth | nprune: {2, …, min(200, 2p+1)}; degree: {1, 2} (1 = additive; 2 = pairwise interactions) |
|  | K-Nearest Neighbors (K-NN) | Makes a prediction for a new experiment based on the results of the most similar past experiments in the training data | knn | k: {5, 7, 9} |
|  | Support Vector Machine (SVM) | Uses a technique called the "kernel trick" to handle highly complex, non-linear relationships in the data | svmLinear | C (cost): {0.25, 0.5, 1.0}; linear kernel |
|  | Artificial Neural Networks (ANN) | Simulates the structure of the human brain to learn extremely complex, non-linear connections between culture conditions and results | nnet | size: fixed at 10 hidden units;  decay: {0, 0.1, 1×10⁻⁴}; single hidden layer; MaxNWts = (p+1)×10 + 11 + 500 |

**Note:** Abbreviations: p = number of input predictors; mtry = variables sampled per split; ntree = number of trees; nrounds = boosting rounds; eta = learning rate; C = cost parameter; ncomp = latent components;
alpha = mixing parameter; lambda = regularization; k = neighbours; size = hidden units; decay = L2 weight regularization; nprune = maximum model terms.

**Table S2 – Optimization algorithms in ADAM**

***Table S2:*** *ADAM offers four optimization algorithms and a brief explanation of the type of optimization, the use and key features when applied in plant tissue culture. Default parameter values are extracted from the ADAM implementation and represent the starting configuration presented to users, all of which can be adjusted prior to running the optimization.*

| **Algorithm** | **Type** | **Use case** | **Key features** | **Default parameters** |
| --- | --- | --- | --- | --- |
| Genetic Algorithm (GA) | Single-objective | Evolutionary computation using selection and mutation. Best used for finding the single best combination of factors to maximize a target (e.g., highest multiplication rate). | High exploration; good for complex, rugged landscapes. | Population size (μ): 50; Offspring (λ): 25;  Max generations: 100; Mutation prob.: 0.10; Crossover prob.: 0.70; Mutation strength (σ): 0.10; Tournament size: 2 |
| Particle Swarm Optimization (PSO) | Single-objective | Swarm intelligence where a population (the "swarm") moves through the search space, influenced by the best solutions found individually and collectively. | Highly efficient at rapidly converging on a single optimum in continuous parameter space. | Swarm size: 50; Max iterations: 100; Inertia weight (w): 0.70;  Cognitive factor (c1): 1.4;  Social factor (c2): 1.4; Velocity limit: 0.20 |
| NSGA-II (Non-dominated Sorting Genetic Algorithm II) | Multi-objective | Uses non-dominated sorting and crowding distance to maintain a diverse set of solutions across the Trade-off Space. | Ideal for finding multiple, high-quality, non-conflicting solutions when optimizing two objectives (e.g., shoot length and rooting success). | Population size (μ): 50; Offspring (λ): 50; Max generations: 100; Mutation prob.: 0.20; Crossover prob.: 0.70; SBX distribution index (η): 15; Polynomial mutation η: 25 |
| SMS-EMOA (S-Metric Selection Evolutionary Multi-Objective Algorithm) | Multi-objective | Uses hypervolume-based selection for superior convergence and solution quality, particularly useful in high-dimensional objective spaces. | Advanced selection based on the volume of space covered by the solutions, effective for three or more conflicting objectives. | Population size (μ): 50; Offspring (λ): 1;  Max generations: 200; Mutation prob.: 0.20; Crossover prob.: 1.0 (fixed);  SBX distribution index (η): 15; Polynomial mutation η: 25; HV reference point offset: 10% |

**Note:** Abbreviations: μ = population size; λ = offspring count; prob. = probability; σ = standard deviation for Gaussian mutation; w = inertia weight; c1 = cognitive acceleration coefficient; c2 = social acceleration coefficient; SBX = simulated binary crossover; η = distribution index controlling operator spread;
HV = hypervolume.

**Appendix S3 – Pipeline validation: ML model training and optimization benchmarking using ZDT2 test function**

To validate the optimization capabilities of ADAM, we conducted a comprehensive benchmark using the ZDT2 test function (Zitzler et al. 2000), a well-established multi-objective optimization problem. This benchmark evaluates both single-objective (GA) and multi-objective (NSGA-II) optimization performance under realistic conditions with noise and uncertainty.

**Database**

A synthetic ZDT2 dataset was generated containing 750 samples with 10 independent variables (factors; F) as inputs (F_1_-F_10_) and two response variables (parameters) as outputs in the model (P_1_, P_2_). The dataset incorporated 10% Gaussian noise to simulate realistic experimental conditions. The ZDT2 function is defined as:

- **First objective**: P_1_ = F_1_
- **Second objective**: P_2_ = g × (1 - (P_1_/g)²)
- **Constraint function**: g = 1 + 9 × Σ(F_i_)/(n-1) for i = 2 to n

The theoretical Pareto front follows the curve P_2_ = 1 - P_1_², providing a known benchmark for optimization quality assessment.

**ML training**

The ML training module of ADAM processed the dataset using eight different algorithms (without ESR) in spot-check mode with 4-fold cross-validation (5 repetitions), resulting in 602 observations in train set, 150 observations in validation set and 148 observations in test set (Table S3.1). The training results demonstrated excellent predictive performance:

***Table S3.1:*** ***ML algorithm performance on the ZDT2 benchmark dataset with 10% Gaussian noise****. Performance metrics (R², RMSE, MAE) are shown for both objective functions (P1 and P2) across training, cross-validation (CV), and test sets. Bold values indicate the best performance for each metric.*

| **Algorithm** | **Target** | | **CV R²** | **Train R²** | **Test R²** | **CV RMSE** | **Train RMSE** | | **Test RMSE** | **Test MAE** |  |
| --- | --- | --- | --- | --- | --- | --- | --- | --- | --- | --- | --- |
| **Elastic Net*** | P_1_ | **0.969±0.004** | | 0.969 | **0.975** | 0.057**±**0.004 | | 0.057 | **0.050** | **0.035** |  |
| SVM | P_1_ | 0.968**±**0.004 | | 0.969 | **0.975** | 0.057**±**0.004 | | 0.056 | **0.050** | **0.035** |  |
| PLS | P_1_ | **0.969±0.004** | | 0.970 | 0.974 | 0.057**±**0.004 | | 0.056 | 0.051 | **0.035** |  |
| Neural network | P_1_ | 0.968**±**0.005 | | 0.973 | 0.973 | 0.057**±**0.005 | | 0.053 | 0.052 | 0.036 |  |
| MARS | P_1_ | 0.969**±**0.004 | | 0.970 | 0.973 | **0.056±0.004** | | 0.056 | 0.052 | **0.035** |  |
| Random Forest | P_1_ | 0.966**±**0.005 | | **0.994** | 0.969 | 0.059**±**0.005 | | **0.024** | 0.055 | 0.038 |  |
| kNN | P_1_ | 0.868**±**0.019 | | 0.892 | 0.876 | 0.120**±**0.007 | | 0.105 | 0.111 | 0.080 |  |
| **SVM*** | P_2_ | 0.962**±**0.005 | | 0.963 | **0.952** | 0.416**±**0.029 | | 0.406 | **0.446** | 0.317 |  |
| PLS | P_2_ | **0.963±0.005** | | 0.963 | 0.951 | **0.412±0.025** | | 0.406 | 0.448 | **0.316** |  |
| ENet | P_2_ | 0.962**±**0.005 | | 0.964 | 0.951 | 0.416**±**0.026 | | 0.404 | 0.449 | 0.319 |  |
| MARS | P_2_ | 0.959**±**0.005 | | 0.964 | 0.949 | 0.434**±**0.024 | | 0.404 | 0.461 | 0.323 |  |
| Neural network | P_2_ | 0.962**±**0.005 | | 0.968 | 0.947 | 0.418**±**0.026 | | 0.379 | 0.469 | 0.321 |  |
| Random Forest | P_2_ | 0.947**±**0.006 | | **0.989** | 0.942 | 0.512**±**0.037 | | **0.224** | 0.488 | 0.341 |  |
| kNN | P_2_ | 0.939**±**0.006 | | 0.957 | 0.937 | 0.531**±**0.032 | | 0.442 | 0.510 | 0.350 |  |

*Note: Elastic Net and SVM was chosen for GA and NSGAII (based on best Test R² and Test RMSE metrics) to demonstrate the optimization performance on ZDT2_10_noise data set.

For the first response variable (parameter P_1_) most algorithms (Elastic Net, SVM, PLS, Neural Network, MARS), the Train R^2^ and Test R*^2^* are very similar and over 0.96, indicating that the model can explain over 96% of the variability in the data for this parameter. This is the most critical sign of a robust model. It means the models have learned the underlying patterns rather than simply memorizing the training data (as explained above).

For the second response variable (parameter P_2_), the models also perform very well on predicting R^2^ values over 0.94, which is still an excellent result.

**Optimization I – Single-objective approach with GA**

To demonstrate the genetic algorithm as a single-objective optimization on the ZDT2 dataset, we set P_2_ as the target for minimization using SVM from Table S3.1. The configuration utilized a population size of 100 individuals with 50 offspring generated per generation, running for a total of 250 generations. Key algorithmic parameters included a mutation probability of 10%, a crossover probability of 70%, and tournament selection with a tournament size of 2 individuals. Boundaries for x_1_-x_10_ were set to range from 0 to 1.

The genetic algorithm successfully optimized the P_2_ objective function, achieving substantial improvement from an initial best fitness of 7.579 to a final best of 0.481, representing a 93.7% reduction in the objective value (Figure S3.2). The algorithm demonstrated complete convergence with zero fitness change in the final 10 generations, indicating that the optimization process reached a stable solution. The convergence of both best and mean fitness to identical values (0.481) suggests the entire population converged to the same optimal solution.


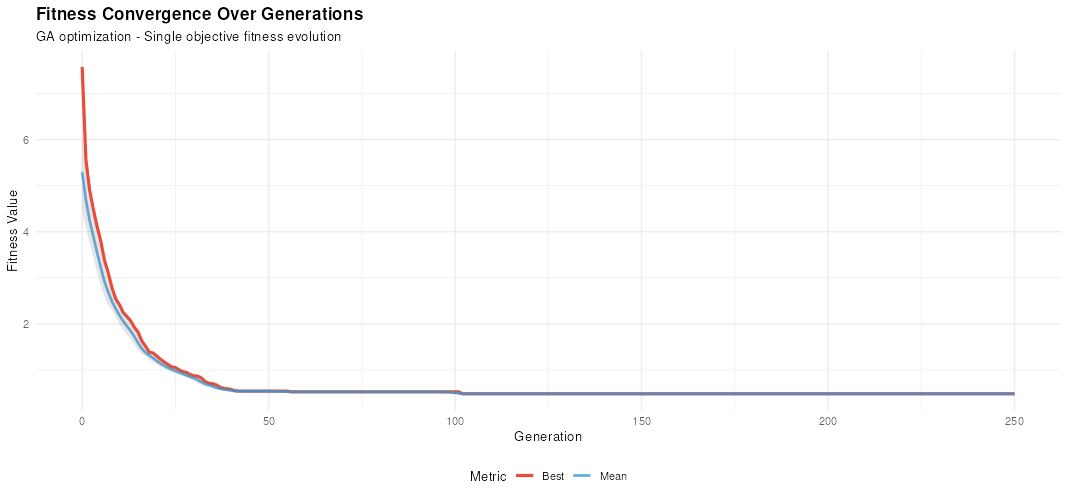
 **Figure S3.2: Optimization performance of SVM-GA on ZDT2_10_noise while minimizing P_2_.** Y-axis shows raw fitness value of P_2_ as (Best: lowest, Mean: average of each population) against number of generations on x-axis. This figure was captured from the ADAM application interface while monitoring optimization performance.

The SVM-GA optimization on the ZDT2 problem successfully identified the optimal solution with F_1_=1, F_2_-F_10_=0, achieving a final objective value of P_2_=0.4808. This solution aligns with the theoretical optimum for the ZDT2 test function, where the global minimum occurs when the first variable is maximized (F_1_=1) and all remaining variables are set to zero.

**Optimization II – Multi-objective approach with NSGAII**

To demonstrate multi-objective optimization capabilities, we employed the Non-dominated Sorting Genetic Algorithm II (NSGA-II) on the ZDT2 dataset, simultaneously minimizing both P_1_ and P_2_ objectives using ENet (P_1_) and SVM (P_2_) models from Table S3.1. The configuration utilized a population size of 100 individuals with 100 offspring generated per generation, running for a total of 100 generations and conducting 10,100 function evaluations. Key algorithmic parameters included a mutation probability of 20%, a crossover probability of 70%, simulated binary crossover (SBX) with η=15, and polynomial mutation with η=25. Boundaries for F_1_-F_10_ were set to range from 0 to 1.

The NSGA-II algorithm successfully explored the multi-objective trade-off space (Figure S3.3A,B), demonstrating characteristic Pareto front evolution over the optimization process. The algorithm showed steady improvement in both objectives, with the best fitness progressing from an initial value of 1.504 to a final best of 1.929, representing a 28.3% improvement in the normalized fitness value. The mean population fitness improved from 0.993 to 1.681, indicating consistent population-wide advancement toward the Pareto optimal region. Despite reaching the maximum generation limit, the algorithm maintained active search behavior with a convergence measure of 0.002277 in the final 10 generations, suggesting potential for further optimization given additional computational budget.


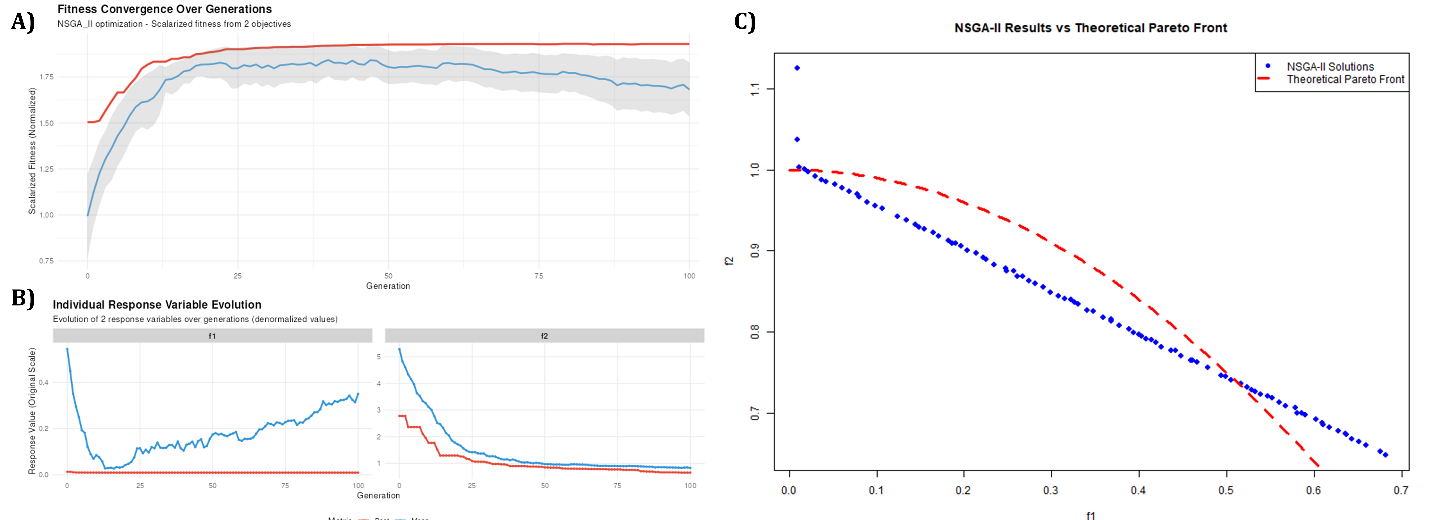


**Figure S3.3: Optimization performance of ENET-SVM-NSGAII on ZDT2_10_noise while minimizing P_1_ and P_2_.** A) Y-axis shows scalarized fitness value (calculated as the cumulative sum of normalized objectives) of P_1_ and P_2_ as (Best: lowest, Mean: average of each population) against number of generations on x-axis. B) Raw fitness values for the two objective variables P_1_ and P_2_. C) Found final pareto front vs. theoretical optimal pareto front This figure was captured from the ADAM application interface while monitoring optimization performance.

The NSGA-II optimization on the ZDT2 problem successfully identified a diverse Pareto front comprising 100 non-dominated solutions. The optimization explored the decision variable space with F_1_ ranging from 0.002 to 0.684, while variables F_2_-F_10_ remained close to zero with maximum values not exceeding 0.051. This resulted in objective function values spanning P_1_ from 0.009 to 0.682 and P_2_ from 0.648 to 11.254, effectively capturing the trade-off relationship between the competing objectives.

The Pareto front approximation shows good coverage of the feasible objective space, though some deviation from the theoretical optimum is observed (Figure S3.3C). This variance can be attributed to the inherent approximation limitations of the ENET and SVM ML models (R²=0.975 for P_1_ and R²=0.952 for P_2_), computational constraints with 100 generations, and the stochastic nature of the evolutionary search process. Despite these factors, the NSGA-II successfully demonstrated its capability to identify multiple trade-off solutions, providing decision-makers with a comprehensive set of Pareto optimal alternatives for the ZDT2 benchmark problem.
